# Modeling of Metalized Food Packaging Plastics Pyrolysis Kinetics Using an Independent Parallel Reactions Kinetic Model

**DOI:** 10.3390/polym12081763

**Published:** 2020-08-06

**Authors:** Samy Yousef, Justas Eimontas, Nerijus Striūgas, Mohammed Ali Abdelnaby

**Affiliations:** 1 Department of Production Engineering, Faculty of Mechanical Engineering and Design, Kaunas University of Technology, LT-51424 Kaunas, Lithuania; 2 Department of Materials Science, South Ural State University, Lenin prospect 76, 454080 Chelyabinsk, Russia; 3 Lithuanian Energy Institute, Laboratory of Combustion Processes, Breslaujos 3, LT-44403 Kaunas, Lithuania; justas.eimontas@lei.lt (J.E.); Nerijus.Striugas@lei.lt (N.S.); 4 Department of Production Engineering and Printing Technology, Akhbar Elyom Academy, 6th of October 12566, Egypt; Muhmmad.aly@akhbaracademy.edu.eg

**Keywords:** plastic waste, metalized food packaging plastics, pyrolysis treatment, independent parallel reaction kinetic model

## Abstract

Recently, a pyrolysis process has been adapted as an emerging technology to convert metalized food packaging plastics waste (MFPWs) into energy products with a high economic benefit. In order to upscale this technology, the knowledge of the pyrolysis kinetic of MFPWs is needed and studying these parameters using free methods is not sufficient to describe the last stages of pyrolysis. For a better understanding of MFPWs pyrolysis kinetics, independent parallel reactions (IPR) kinetic model and its modification model (MIPR) were used in the present research to describe the kinetic parameters of MFPWs pyrolysis at different heating rates (5–30 °C min^−1^). The IPR and MIPR models were built according to thermogravimetric (TG)-Fourier-transform infrared spectroscopy (FTIR)-gas chromatography−mass spectrometry (GC-MS) results of three different types of MFPWs (coffee, chips, and chocolate) and their mixture. The accuracy of the developed kinetic models was evaluated by comparing the conformity of the DTG experimental results to the data calculated using IPR and MIPR models. The results showed that the dependence of the pre-exponential factor on the heating rate (as in the case of MIPR model) led to better conformity results with high predictability of kinetic parameters with an average deviation of 2.35% (with an improvement of 73%, when compared to the IPR model). Additionally, the values of activation energy and pre-exponential factor were calculated using the MIPR model and estimated at 294 kJ mol^−1^ and 5.77 × 10^17^ kJ mol^−1^ (for the mixed MFPW sample), respectively. Finally, GC-MS results illustrated that pentane (13.8%) and 2,4-dimethyl-1-heptene isopropylcyclobutane (44.31%) represent the main compounds in the released volatile products at the maximum decomposition temperature.

## 1. Introduction

Metalized food packaging plastic waste (MFPWs) is classified as a plastic waste with a very complex composition [1]. As it consists of a number of polymeric and aluminum layers (e.g., PE, PP, PVC, PS, PET, etc.) joined mechanically and chemically by air emptying [2,3], this improves their performance and addresses such environmental factors as moisture and sunlight [4]. According to the life cycle evaluation of MFPWs that was conducted by many studies [5,6], once it ends its very short service period, which is estimated for a few days, it is difficult and not profitable to separate these layers and to recover them in the shape of secondary raw materials using the traditional mechanical and chemical practices such as grinding and dissolution treatment [3,7,8]. Additionally, these studies demonstrated clearly that the separation of MFPWs layers is always a great challenge as the surface area of the thinner materials is higher in comparison to the bulk material and it is exposed more to the atmospheric oxygen [9], thus MFPWs mostly end in landfilling, followed by incineration with other municipal solid waste, and the rate of recycling is almost negligible, being around~20% [10,11].

Additionally, some studies were performed to recover Al, but these studies did not receive much attention as they were focused on the metallic part only, without considering the polymeric part which represents the biggest part containing a lot of toxic materials [12,13]. In order to recycle all fractions, Samy et al. (2019) used a thermal disengagement process to transform thermally the polymer–metal packaging materials into considerably pure metal and graphitic carbon particles in presence of Argon gas followed by grinding to recycle the materials into Al particles. Although the results were very promising but still in the earlier stages and missing economic evaluation [14,15]. Since MFPWs contain a lot of flammable materials in the polymer fraction, thermal treatments started gaining a lot of attention to convert this fraction into higher added-value energy products using gasification and pyrolysis processes [16,17]. The studies showed that the resulting values from the pyrolysis process are much higher when compared to the gasification process, where the pyrolysis process helps to convert the plastic to liquid oil with a yield in the range of 14–60% based on the composition [18,19,20]. Under the thermal treatment in a nitrogen ambient, the polymer part decomposes into volatiles, gases, and char products [21]. Therefore, the pyrolysis process remains the closest technique to reality, and has made promising results in terms of yield, interest, economics, and emissions [22,23]. Furthermore, the knowledge of kinetics for thermal decomposition of MFPWs is very important for the design of pyrolysis reactors, in order to select the optimum design variables, including material selection, safety factors, applied stress at the beginning and end of the reaction, emission gases, etc., thus to determine the thickness of reactor shell and other geometries in future industrial practices [24,25].

Generally, the pyrolysis kinetics of mixed plastic waste, including plastic films and MFPWs is described as a complex process consisting of many heterogeneous reactions that can be classified as a primary parallel, secondary parallel, and competitive reactions, resulting in several layers with different composition and different stacking sequences of layers. Thus, it is difficult to suggest mechanisms for accurate characterization of their decomposition, as well as their pyrolysis kinetics and consequently order of decomposition reactions [26,27]. Therefore, the selection of the most appropriate kinetic model for the thermal decomposition of plastic waste is still a controversial topic in the scientific community. Besides, these kinetic models can be classified into three main categories: the single reaction model (SRM), the consecutive reaction model (CRM), and the independent parallel reaction (IPR) model. SRM is a very simple method and inadequate for describing the latter phases of the pyrolysis process, while the inaccurate assumption of CRM which was built on the decomposition of one subcomponent at the beginning of the reaction until its end, followed by degradation of the second element, etc., may lead to implausible kinetic results [28,29]. Meanwhile, if it is assumed that all pseudocomponents are degraded individually and simultaneously, the IPR model seems to be more accurate to describe the kinetics of pyrolysis of MFPWs, especially when this waste contains a lot of layers with almost the same organic compounds ensuring simultaneous decomposition of all elements [30].

Although the kinetics of pyrolysis packaging plastics (PS/PP, PET/PP, PET/PE film and PP/PE film) was studied thoroughly using different kinetics models [31,32,33], only one study was focused on pyrolysis kinetics of MFPWs using model-free methods (as the simplified formal reaction model) [11], which resulted in big variation in the kinetic parameters at the lower conversion rate (up to 0.7) and unreasonable kinetic parameters at the higher conversion rate. Additionally, it was assumed that by using this model each peak in the DTG analysis curves corresponds to the individual and sequential decomposition of the pseudocomponents, giving the impression that no interactions occur between them [34]. In order to create more precise pyrolysis kinetics of MFPWs and to decrease variation between experimental and calculated data, the IPR model was used in the present research to describe the DTG behavior of MFPWs at different heating rates. Additionally, the modified IPR model was used to eliminate the aforementioned drawbacks of previous investigations as far as possible and to decrease the variation.

## 2. Experimental

### 2.1. Materials Selection and Analysis

In order to ensure diversity and to increase the accuracy of results obtained from the developed pyrolysis kinetic model, the experiments were performed on three different types of metalized food packaging plastic waste (MFPWs): coffee, chips, and chocolate and their mixture. Firstly, the selected samples were milled into fine particles using a coffee grinder followed by sieving and choosing the smallest particle to minimize heat transfer resistances and mass-to-surface volume ratio. Moisture, volatiles, and ash contents of the milled MFPW samples and their mixture were measured according to the ASTM (E1756–01, E872–82, and E1755–01) standard methods using the proximate analysis (CHNS/O 2400 PerkinElmer) [35]. Additionally, carbon, hydrogen, nitrogen, oxygen, and sulfur contents were measured using an elemental analyzer.

### 2.2. Thermogravimetric Analysis

The thermal decomposition experiments on the selected samples were performed in a thermogravimetric analyzer TGA (NETZSCH STA 449 F3 Jupiter analyzers). The experiments were carried out on approximately 10–15 mg of each patch. The selected samples were placed in the TGA microbalance pan, then the furnace was heated from room temperature up to 900 °C at different heating rates (5, 10, 15, 20, 25, and 30 °C min^−1^) under N_2_ atmosphere at a flow rate of 50 mL min^−1^. Then sample temperature was measured (with a thermocouple attached directly to the crucible) and the weight loss and the derivative of each sample were recorded using TGA software and DTG analysis. Additionally, the effect of heating rates on pyrolysis characteristics and the devolatilization index (Di) of volatile matters released from the mixed MFPW sample was investigated and calculated using Equation (1) [36]:

(1) $$D_{i}=\frac{R_{max}}{T_{i}T_{m}\;\Delta T_{1/2}}$$

where *R_max_*, *T_i_*, and *T_m_*, represent the mass loss rate, the initial temperature, and the temperature at a maximum degradation rate, respectively, and can be obtained from TGA curves and DTG peaks. *ΔT_1/2_* is defined as the temperature range at *R_d_*/*R_max_* = 0.5, where *R_d_* represents the decomposition rate and can be generated from DTG curves.

### 2.3. Chemical Analysis of the Formulated Chemical Compounds

Fourier-transform infrared spectroscopy (FTIR: PerkinElmer-L1280127) and gas chromatography-mass spectrometry coupled with thermogravimetry (TG-GC-MS, Thermo Scientific ISQ™ single quadrupole GC-MS system) were employed to analyze the released volatile products from the mixture sample at the maximum decomposition rates (based on DTG results) in the range of 30–600 m/z by collecting the generated gas at these specified temperatures using an Automation Autoinjector™ module connected with the TGA system; then they were examined using GC−MS [37].

### 2.4. Kinetic Models of MFPWs Pyrolysis

MFPWs are mainly composed of three subcomponents: polymers, dyes, and Al layer; all these pseudocomponents are degraded individually in the same temperature range, ensuring a possibly simultaneous decomposition. Therefore, the IPR model and its modification were used as the most accurate to describe the kinetics of pyrolysis. Additionally, the rate of weight loss is calculated considering the individual reaction rates and their respective mass fraction.

#### 2.4.1. Independent Parallel Reactions Kinetic Model

According to the IPR model, main pseudocomponents of MFPWs (plastic layers, dyes, and Al layer) decompose individually and simultaneously in the form of three or more independent parallel first or n^th^ order reactions into gases, volatiles, and char. In this case, the primary conversion (*X*) of MFPW samples can be calculated using Equation (2) based on the results of thermogravimetric (TGA) experiments, while individual dependence of conversion rate on the temperature of each subcomponent (*i*) can be described as indicated in Equation (3) Meanwhile, the overall rate of conversion (*X*) is a linear combination of the rates of partial reactions (Equation (4)), considering the mass fraction of each of three subcomponents (*c_i_*), thus, the rate of mass loss can be determined by using Equation (5)

(2) $$X=\frac{m_{o}-m_{t}}{m_{t}-m_{f}}$$

(3) $$\frac{{\mathrm{dX}}_{i}}{\mathrm{dt}}={\;A}_{i}\exp\;\left(\frac{-E_{\mathrm{ai}}}{\mathrm{RT}}\right){\left(1-X_{i}\right)}^{n_{i}}$$

(4) $$\frac{dX}{dt}=-\overset{3}{\underset{i=1}{\sum }}.C_{i}\frac{dX_{i}}{dt}$$

(5) $${\frac{dm}{dt}}^{calc}=-\left(m_{0}-m\right)\overset{3}{\underset{i=1}{\sum }}.C_{i}\frac{dX_{i}}{dt}$$

In order to determine the optimal parameters (*E_i_, A_i_, C_i_*), an algorithm in Matlab^®^ code was built to obtain these parameters for minimized Derivative Thermogravimetry (DTG) objective function. In this case $O.F._{DTG}$ can be described using Equation (6). The gradient-based minimization function fmincon of Matlab^®^ was used in the optimization process to determine the unknown parameters of the developed model. These types of pyrolysis kinetic models need initial values of *E_i_* and *A_i_* for each sample at each heating rate and these values were unknown at the beginning of the running model. In order to decrease the variation of the results and to save running time, the values of *E_i_* (211.31 KJ.mol^−1^) and *A_i_* (2.06342×10^16^ KJ.mol^−1^), which were obtained by Yao et al. (2020) were used in the presented research as an initial suggestion [29]. Finally, the deviation (*Dev.)* between the experimental and calculated curves of DTG curves at the optimal set of parameters was assessed for each experiment using Equation (7) All effective parameters in the developed IPR and MIPR models are described in Table 1.

(6) $$O.F._{DTG}=\overset{N}{\underset{j=1}{\sum }}{\left({\left(\frac{dm}{dt}\right)}_{j}^{obs}-{\left(\frac{dm}{dt}\right)}_{j}^{calc}\right)}^{2}.$$

(7) $$\mathrm{Dev}.(\%)=\frac{100\sqrt{F.O._{DTG}\left(Z-N\right)}}{\max\left(\left|dm/dt\right|\right)}$$

#### 2.4.2. Modified Independent Parallel Reactions Kinetic Model

As shown in the results section, the results determined by the IRP model in terms of deviation were not satisfactory in almost all the cases, in an attempt to develop a model capable of predicting conversion data at any given temperature program with a minimum value of deviations, so Sfakiotakis et al. (2015) modified the IPR model by keeping *E_i_* constant and allowing *A_i_* to vary when heating rate changes as shown in Equation (8) [30].

(8) $$ln(\frac{A_{i}}{A_{oi}})=g_{i}.ln(HR)$$

where *A_oi_* is the pre-exponential factor at a minimum heating rate (5 °C/min in the present case), HR is the nonzero heating rate, and $g_{i}$ is a parameter, which describes the dependence of *A_i_* on the heating rate.

## 3. Results and Discussions

### 3.1. Elemental and Proximate Analyses

Table 2 presents the results of the elemental and proximate values of MFPWs. It is clear that MFPWs contain an appreciable amount of carbon and hydrogen, which can contribute to an increase in the energy value of the synthesized fuel, as a result of higher energy contained in carbon–carbon bonds [38]. As revealed by the elemental analysis in the table, the nitrogen content (0.58%) was lower than that usually found in several other types of plastic wastes, such as: wheat PP (2.34%), PE (2.53%), and PH (2.64%) [39]. This low value of N_2_ and S (0.01 wt %) is desirable in biofuels, which can contribute to the reduction of nitrogen oxides (NOx) and SO_2_ toxic emissions during the conversion process [40]. Additionally, high content of C (81.56%) and O (3.91%) was observed in all the MFPW samples, which indicates that the MFPW samples are a very rich source of carbon precursor that facilitates the conversion process. It is worth mentioning that oxygen content was determined using the analyzer directly. Besides, the proximate analysis results show a significant volatile matter content in the tested samples (87.01%) and low ash content (2.52%). This lower ash content can act as a catalyst for the pyrolysis treatment to promote secondary reactions of volatile decomposition and char formation [35]. The volatile matter for the residues of these MFPWs was higher than the value found by other residues of plastic waste [41]. Thus, these MFPWs could have a relevant potential in energy generation with little variation due to changes in chemical composition and amount of aluminum in each sample.

### 3.2. Thermogravimetric Analysis

**Figure 1:**
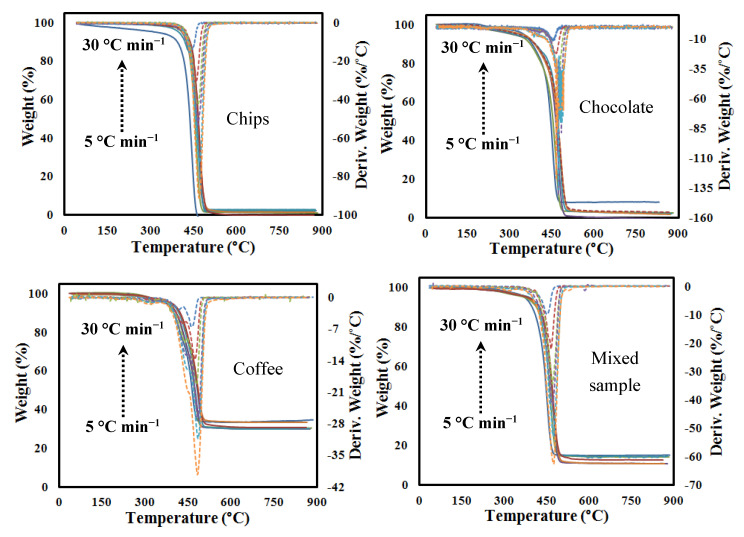
Experimental TGA (solid lines) and DTG (dotted lines) curves at the different heating rates of MFPW samples.

Figure 1 shows the curves of weight loss (TG-solid lines) and the rate of mass loss (DTG-dotted lines) of all selected MFPW samples, at heating rates of 5, 10, 15, 20, 25, and 30 °C min^−1^. It seems that all MFPW samples had similar degeneration features with average total weight loss estimated at 97–99 wt %, except for the coffee sample, the weight loss of which was estimated at 71% due to a higher percentage of Al that prevents decomposition by pyrolysis process. Additionally, it is clear that the removal of the moisture content occurred in temperature ranges of 30–380 °C (~3 wt %). The main decomposition phase with a significant weight loss estimated at 68–95% happens at the temperature ranges of 381–510 °C, with only a sharp peak, as shown in DTG corresponding to the maximum decomposition of all subcomponent of MFPWs at 450 °C into simple organic and inorganic molecules [42].

This means that all polymers and organic components degraded together in the form of a single reaction. Al is a metallic component that cannot decompose even at high temperatures; however, Al can react with the generated gases during the conversion process and some of them can be used as a catalyst (self-catalysts) to accelerate the reaction and to upgrade the obtained fuel [40,43]. Meanwhile, the excess fraction of Al can be kept (undecomposed Al) as a mixture with char devolatilization in the last phase in a wider range of temperatures (502–900 °C) with an average weight loss ~2 wt % for all samples. Finally, it was noted that the maximum rate of pyrolysis increases, with increasing heating rate, as an increasing heat flux can contribute to the penetration of heat inside the sample and increasing of the conversion rate, decrease in exposure time, and acceleration of the degradation process [44]. These results strengthen the principle of that the MFPWs are rich in volatile elements and all of them decompose individually and simultaneously in the form of single peak reaction, while the IPR model is the most accurate to describe the kinetics of pyrolysis of the whole MFPWs conversion process, as shown in detail in the following sections. Finally, the effect of heating rate on pyrolysis characteristics of MFPW samples, including the values of Di, the residue mass (*M_f_*), the final temperature (*T_f_*), *R_max_*, etc., are presented in Table 3. As it can be seen, there exists a slightly increasing trend of volatiles percentage as a function of heating rate for all the samples, because high heat fluxes in the high heating regime reduce the viscosity of the melted solid material, at the same time intensifying the reactions forming volatiles. As the trend is low for the range of 5–30 °C min^−1^, the kinetic models in this study do not take into account a variation of the coefficient *C_i_* as a function of heating rate.

### 3.3. Chemical Analysis of the Synthesized Chemical Compounds Using FTIR and GC-MS

**Figure 2:**
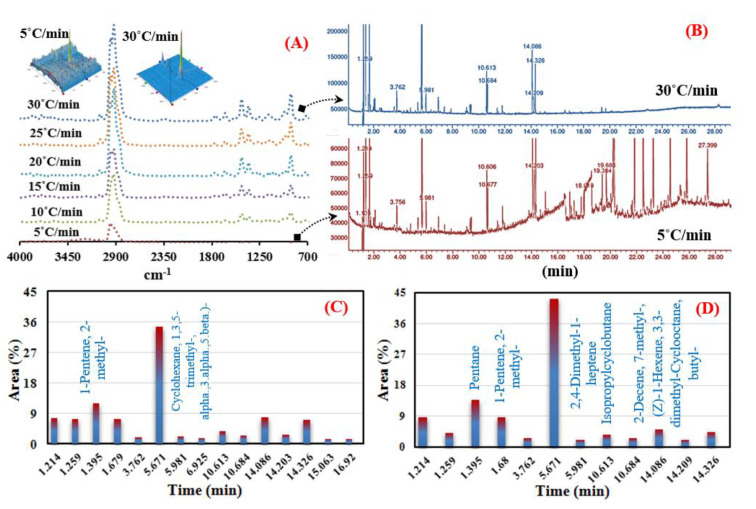
(**A**) 2D-3D FTIR analysis, (**B**) GC-MS analysis at 5 and 30 °C/min, and (**C**,**D**) the obtained GC-MS compounds at 5 and 30 °C min^−1^.

The effect of heating rates (5–30 °C.min^−1^) on the synthesized chemical compounds at the maximum decomposition temperatures (336–340 °C based on DTG results) was initially examined using FTIR-TG. The observation process using FTIR and GC-MS was performed on the mixture MFPW sample only (which represents the real case). Figure 2A shows a 2D–3D FTIR spectra analysis of the mixed MFPW sample under the specified conditions. It seems that at the lowest heating rate (5 °C min^−1^), Methane and carboxylic acid residue was observed only at 2950 cm^−1^ with weak absorbance, as heating rates increasing the absorbance of this peak increased significantly and other two weak peaks appeared at 870 cm^−1^ (corresponding to C–O−C stretching) and 1460 cm^−1^ (–CH2–bending vibration) as a result of increased heat flux which led to an increase in the conversion rate and decomposition of all organic components of MFPW layers [45].

In order to indicate the main compounds of the released volatile products synthesized at the specified decomposition temperatures and heating rates of 450 °C (5 °C min^−1^) and 476 °C (30 °C min^−1^) more precisely, GC-MS analysis was used to examine the synthesized chemical compounds at low and high heating rates (5 and 30 °C min^−1^). Figure 2B shows the GC-MS analysis of the mixed MFPW sample at 5 and 30 °C min^−1^. As shown in the GC-MS analysis, several compounds were observed at the lowest heating rate (5 °C min^−1^), including propene (7.4%), pentene (12%), heptane (7.3%), cyclohexane (35%), cyclododecanemethanol (7%), etc. (Figure 2C). When the heating rate was increased to 30 °C min^−1^, the obtained GC compounds of the mixed sample increased significantly (Figure 2D), especially Pentane (13.8%) and 2,4–Dimethyl−1–heptene Isopropylcyclobutane (44.31%) and became the most abundant pyrolysis products due to the significant increase in the generated heat flux [17,46]. Additionally, this is due to the complexity of the feedstock which leads to that the intensity of the released volatile products at the lowest and highest heating rates was lower than the GC detection limit, thus generating several overloaded peaks, which makes it hard to find matched compounds for these obtained peaks. These pyrolysis compounds can be used in many applications related to the production of chemicals, fungicide Propiconazole, stabilizers, cleaners, pharmaceuticals, etc. [47]. Finally, and for a better understanding of molecular mechanisms that take place during the thermal conversion of MFPWs, the IPR and MIPR reaction models were applied and all the results are explained in detail in the following sections.

### 3.4. Kinetic Evaluation of the Pyrolysis of MFPWs

The effect of heating rates on the pyrolysis kinetics behavior of MFPWs was investigated in this section using the IPR and MIPR reaction models, and activation energies at a certain conversion degree and pre-exponential factor were evaluated. Additionally, the deviation between the observed and fitting DTG curves was calculated to assess the performance results of each method.

### 3.5. IPR Kinetic Model

**Figure 3:**
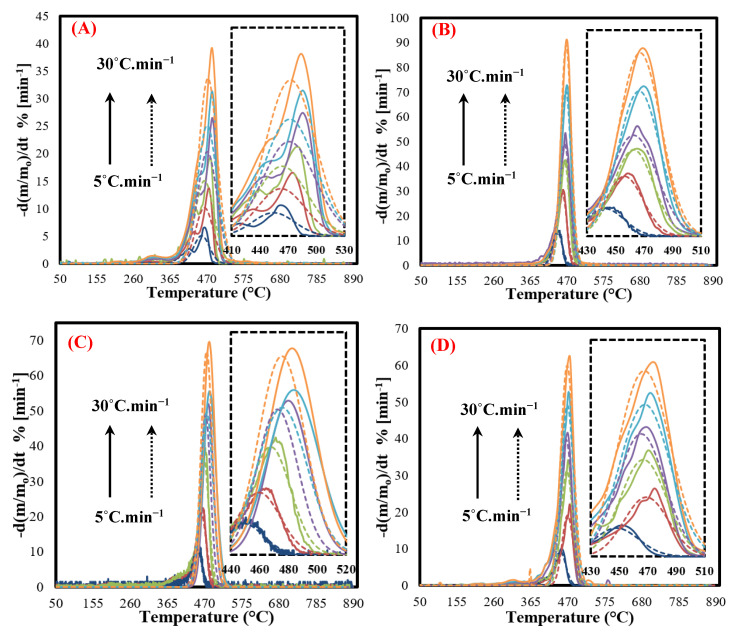
Experimental (solid lines) and calculated results (dotted lines) of the IPR model at heating rates 5–30 °C min^−1^ for (**A**) coffee, (**B**) chips, (**C**) chocolate, and (**D**) and mixed sample.

Based on the results obtained by Mumladze et al. (2018), MFPWs contain more than three pseudocomponents, including dye layer (pigments and organic component), various plastic films (like PE, PP, PVC, PS, PET, etc.), and a metallic layer (Al) [7,23]. As observed in the DTG curves in Section 3.2, the curves consist of two visible peaks resulting from simultaneously decomposed pseudocomponents in two forms, the organic part in dye layers and sealing and plastic films [48], while Al layer cannot decompose due to its high melting temperature > 800 °C. These two peaks cannot be simulated using a single reaction model [29], therefore IPR was employed for that propose. Figure 3 shows the experimental (solid lines) and simulated (dotted lines) curves of DTG at all heating rates of 5–30 °C min^−1^ for all the MFPW samples using Equations (4) and (5). As shown in the figures, the kinetic parameters derived from the calculated conversion data to the experimental data were noted to be invalid for precise prediction of conversion. Meanwhile, the fitted curves differed quite fairly from the experimental data. They matched poorly, in particular, the simulated curves contain a single peak, while the experimental data contain two peaks corresponding to heating rates. Additionally, the simultaneous fit of the simulated data to the experimental data was not satisfactory in all the MFPW samples and big deviation in the range 7% (chips) to 12% (coffee) was observed, what means that IPR can give unacceptable results for kinetic behavior of the pyrolysis of MFPWs under different heating conditions. These big deviations in the IPR results were caused because this type of waste (MFPWs) contains a greater number of pseudocomponents and pseudosubcomponents and this model is recommended in the scenario that does not have more than four components (including pseudosubcomponents). These results are consistent with the data reported in the literature of the pyrolysis kinetics of mixed plastic waste and confirm that MFPWs contain more than four components (multilayers) [43].

### 3.6. Modified IPR Kinetic Model

**Figure 4:**
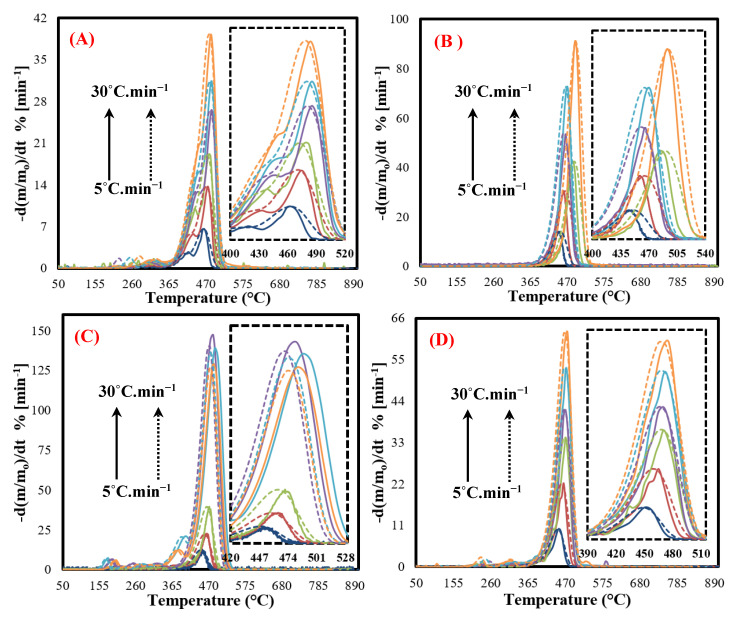
Experimental and calculated results of the modified IPR model at heating rates 5–30 °C/min for (**A**) coffee, (**B**) chips, (**C**) chocolate, and (**D**) and mixed sample.

The MIPR model was used to decrease the deviation obtained from the IPR model and to improve the simultaneous fit of the simulated data to the experimental data of MFPWs, particularly fitting of the main decomposition peaks. Figure 4 shows the simultaneous fit of the calculated data (dotted lines) to the experimental data (solid lines), using the MIPR model, with regard to the curves of DTG for all the studied samples at all heating rates of 5–30 °C min^−1^. As shown in the figure, the decomposition domains (the shape and width) of the pseudocomponents received from the MIPR model are matched with the partial devolatilization curves of all the MFPW samples at all heating rates. It is clear that the MIPR model predicted the DTG experimental results well-matched for all the MFPW samples with deviation estimated at 2.35%. These results can be considered as promising results comparable with the obtained deviation from the distributed activation energy model (DAEM), which was 2.75% (with an improvement of 17%) [49].

Additionally, the mass fraction of subcomponents (c1, c2, and c3) and other kinetic parameters of MFPW pyrolysis, including activation energies and pre-exponential factors, were estimated for all the sets, as illustrated in Table 4. It is worth mentioning that the calculated activation energy and pre-exponential factors for each sample were divided into two components; *E_1_* and *E_2_* (activation energies for the weak and strong peaks, respectively), and *A_1_* and *A_2_* (pre-exponential factors for the weak and strong peaks, respectively). Since it was hard to estimate all these parameters for all samples at all different heating rates, therefore, these parameters were calculated at the highest heating rate (30 °C min^−1^) only, where the constraints of optimum conditions were considered with regard to this heating rate by achieving the highest amount of volatile products (based on GC-MS analysis, as shown in Section 3.3). It seems that the *E_1_* values of the pseudocomponents of all MFPW samples varied in a relatively narrow range from 134 kJ mol^−1^ to 163 kJ mol^−1^. Based on the position of these peaks on the DTG curves (410–435 °C), these weak peaks corresponded to the degeneration of Low-density Polyethylene (LDPE), High-density Polyethylene (HDPE) and Polypropylene (PP) [25]. Additionally, the activation energies of these films were located in the range from 142 kJ mol^−1^ to 164 kJ mol^−1^ [50,51]. Meanwhile, the activation energies of the strong peaks (440–470 °C) were located in the range from 241 to 294 kJ mol^−1^. These strong degradation peaks corresponded to the decomposition of other plastic films, including PVC, PS, PET, and their mixture [31]. These values are within the range of those reported in the literature on plastic and plastic mixture [31,32,33,52,53].

Based on the reported results in the present research, the MIPR model was proven to be superior to the IPR model, as it manifested better prediction results for stepwise experiments of MFPWs in terms of better fitting results and less deviation, and this result agrees with the results in the literature on other plastic mixtures. Additionally, when compared with the common modeling methods like distributed activation energy model (DAEM), the MIPR model was characterized by high accuracy, where the obtained deviation was estimated by 2.35% (in case of MIPR model) and 2.75% (in case of DAEM). In addition, the MIPR model is characterized by simplicity, faster, and high performance. Therefore, the MIPR model is recommended for use in modeling and evaluation of the kinetics of a multikind waste sample with high chemical complexity, like in the case of metalized food packaging plastics.

## 4. Conclusions

In the present research, the thermal and chemical degradation of some metalized food packaging plastics waste (MFPWs; coffee, chips, chocolate, and its mixture) was studied using the TG-FTIR-GC-MS system at different heating rates. Then all pyrolysis characteristics of volatile matters released from the mixed MFPW sample were studied, and then the pyrolysis kinetics was modeled using the IPR kinetic model and its modification (MIPR). The results showed that the application of the IPR model for simultaneous fitting of the DTG experimental data to the calculated data at heating rates was unsatisfactory for all the MFPW samples with deviations up to 12%. The results indicated the exceptional ability of the MIPR model to perform nonlinear estimation of kinetic parameters of the MFPW pyrolysis under a stepwise heating program with a deviation of 2.35%. Additionally, the kinetic MIPR model showed that the activation energies of the strong peaks (440–470 °C) were estimated in the range from 241 to 294 kJ mol^−1^ due to decomposition of PVC, PS, and PET, while the activation energies of the weak peaks (410–435 °C) were estimated in the range from 134 kJ mol^−1^ to 163 kJ mol^−1^ due to decomposition of PE and PP. Based on the obtained results, the MIPR model is highly recommended to model the kinetic pyrolysis of MFPWs with high prediction and accuracy of results.

## Figures and Tables

**Table 1:** Definition of the effective parameters of the independent parallel reactions (IPR) model [34].

Equation No.	Parameters	Definition
**2**	*m_o_*, *m_t_*, *m_f_*	The initial, instantaneous, and final mass of the sample, respectively
**3**	$X_{i}$, $A_{i}$, *E_i_*, *n_i_*, *t, T, R*	The conversion, pre-exponential factor, activation energy, apparent reaction order of each subcomponent i, time, temperature, and the universal gas constant, respectively.
**4**	*C_i_*	The mass fraction of each of the three subcomponents
**5**	*dm/dt*	The rate of mass loss
**6**	$O.F._{DTG}$	Derivative Thermogravimetry (DTG) objective function
**7**	Z, N	The number of data points and the number of parameters employed in the model, respectively.

**Table 2:** Elemental analysis and proximate analysis of all samples.

Sample Code	Elemental Analysis (wt %)					Proximate Analysis (wt %)			
	C	H	N	S	O	Moisture	Volatile Matter	Fixed Carbon	Ash
MFPW_1_	82.64	14.58	0.74	0.01	3.84	0.23	93.87	4.98	1.07
MFPW_2_	80.75	13.92	0.48	0.02	4.02	0.35	95.03	3.67	1.22
MFPW_3_	81.28	14.81	0.52	0.01	3.86	0.16	72.14	22.54	5.28
**Average**	81.56	14.44	0.58	0.01	3.91	0.25	87.01	10.40	2.52

**Table 3:** The pyrolysis characteristic parameters for metalized food packaging plastics waste (MFPWs) at different heating rates.

Pyrolysis Parameters	Heating Rate (°C min^−1^)					
	5	10	15	20	25	30
**Chips sample**						
*T_i_* (°C)	321.85	408.94	416.13	416.7	418.7	418.8
*T_m_* (°C)	448.4	458.2	465.88	466	469.4	470.4
*T_f_* (°C)	464	494	508	512.37	517.17	564.003
*R_max_* (%/min)	13.8	30.47	42.42	53.44	72.27	91.19
*D_i_* (% min^−1^ °C^−3^)	3.4 × 10^−6^	5.69 × 10^−6^	6.97 × 10^−6^	8.00 × 10^−6^	1.00 × 10^−5^	1.25 × 10^−5^
$\;\Delta T_{1/2}$	28.2	28.586	31.4	34.4	36.6	37.1
*M_f_* (%)	0.073	1.614	2.183	2.92	2.4	0.01
**Chocolate sample**						
*T_i_* (°C)	306.1	306.94	309.4	315.95	338	340.49
*T_m_* (°C)	450.526	466.74	471.12	471.02	472.4	465
*T_f_* (°C)	555.316	557	566.31	573.1	580.91	591
*R_max_* (%/min)	12.6	21.77	39	79.05	80.4	81.78
*D_i_* (% min^−1^ °C^−3^)	3.45 × 10^−6^	4.34 × 10^−6^	9.91 × 10^−6^	3.32 × 10^−5^	2.65 × 10^−5^	2.72 × 10^−5^
$\;\Delta T_{1/2}$	26.457	35	27	16	19	19
*M_f_* (%)	7.9	3.95	0.31	3.1	3.2	3.3
**Coffee sample**						
*T_i_* (°C)	372.72	380.4	381.311	384	392	394.8
*T_m_* (°C)	464.5	475	478.63	485.8	486	486
*T_f_* (°C)	484.8	495.6	502.3	512.81	527.33	585.46
*R_max_* (%/min)	6.5734	15.98	19.2	26.55	31.3792	39.2365
*D_i_* (% min^−1^ °C^−3^)	1.19 × 10^−6^	2.77 × 10^−6^	2.59 × 10^−6^	3.08 × 10^−6^	3.23 × 10^−6^	3.95 × 10^−6^
$\;\Delta T_{1/2}$	31.8	31.95	40.6	46.14	50.9707	51.815
*M_f_* (%)	34.688	30.449	29.604	29.8491	33.2312	30.4352
**The mixed sample**						
*T_i_* (°C)	378	384.67	388	391	384.19	385.19
*T_m_* (°C)	449.64	465	470.05	470.13	471.7	476
*T_f_* (°C)	515	521	537	556	581	593
*R_max_* (%/min)	9.9123	22.1084	34.3	41.65	52.8447	62.04
*D_i_* (% min^−1^ °C^−3^)	1.62 × 10^−6^	3.39 × 10^−6^	5.141 × 10^−6^	5.96 × 10^−6^	7.33 × 10^−6^	8.25 × 10^−6^
$\;\Delta T_{1/2}$	36	36.5	36.6	38	39.8	41
*M_f_* (%)	15.2	14.8	11.29	14.92	11.06	13.07

**Table 4:** The pyrolysis characteristic parameters for MFPW at different heating rates.

Parameter	Chips	Chocolate	Coffee	Mixture
*E*_1_	145.1549	133.9133	143.0858	163.3006
*A*_1_ (at 30 °C/min)	2.86 × 10^13^	1.19 × 10^12^	3.92 × 10^13^	6.44 × 10^14^
c_1_	0.253553	0.297861	0.301881	0.388108
*E*_2_	261.7056	241.4376	257.975	294.4211
*A*_2_ (at 30 °C/min)	2.56 × 10^16^	1.06 × 10^15^	3.51 × 10^16^	5.77 × 10^17^
c_2_	0.554647	0.402139	0.339819	0.411892
**MIPR deviation (%)**	3.122	3.373	2.18	2.35
